# Exosomes derived from pro‐inflammatory bone marrow‐derived mesenchymal stem cells reduce inflammation and myocardial injury via mediating macrophage polarization

**DOI:** 10.1111/jcmm.14635

**Published:** 2019-09-26

**Authors:** Ruqin Xu, Fangcheng Zhang, Renjie Chai, Wenyi Zhou, Ming Hu, Bin Liu, Xuke Chen, Mingke Liu, Qiong Xu, Ningning Liu, Shiming Liu

**Affiliations:** ^1^ Guangzhou Institute of Cardiovascular Disease, Guangdong Key Laboratory of Vascular Diseases, State Key Laboratory of Respiratory Disease, The Second Affiliated Hospital of Guangzhou Medical University Guangzhou Guangdong China

**Keywords:** exosomes, inflammation, macrophage polarization, mesenchymal stem cells, myocardial infarction

## Abstract

Exosomes are served as substitutes for stem cell therapy, playing important roles in mediating heart repair during myocardial infarction injury. Evidence have indicated that lipopolysaccharide (LPS) pre‐conditioning bone marrow‐derived mesenchymal stem cells (BMSCs) and their secreted exosomes promote macrophage polarization and tissue repair in several inflammation diseases; however, it has not been fully elucidated in myocardial infarction (MI). This study aimed to investigate whether LPS‐primed BMSC‐derived exosomes could mediate inflammation and myocardial injury via macrophage polarization after MI. Here, we found that exosomes derived from BMSCs, in both Exo and L‐Exo groups, increased M2 macrophage polarization and decreased M1 macrophage polarization under LPS stimulation, which strongly depressed LPS‐dependent NF‐κB signalling pathway and partly activated the AKT1/AKT2 signalling pathway. Compared with Exo, L‐Exo had superior therapeutic effects on polarizing M2 macrophage in vitro and attenuated the post‐infarction inflammation and cardiomyocyte apoptosis by mediating macrophage polarization in mice MI model. Consequently, we have confidence in the perspective that low concentration of LPS pre‐conditioning BMSC‐derived exosomes may develop into a promising cell‐free treatment strategy for clinical treatment of MI.

## 1 INTRODUCTION

Numerous studies have indicated that the transplantation of mesenchymal stem cells (MSCs) effectively promotes heart repair and potentially prevents heart remodelling after myocardial infarction (MI).^1^, ^2^, ^3^ It has been generally recognized that MSCs have the capacity of self‐renewal and an ability to differentiate into multiple lineages^4^, ^5^; however, poor survival rate of transplanted cells limits the effectiveness of MSC therapy.^6^, ^7^ Recent studies have suggested that MSC‐derived exosomes play an important role in regulating biological function and molecular mechanisms of MSCs,^8^, ^9^ and may serve as alternatives to cell therapy of MSC transplantation in MI.^10^, ^11^, ^12^

Exosomes are the smallest membrane vesicles, to be around 40‐100 nm in diameter,^13^ with cargoes of proteins, lipids, mRNAs and microRNAs (miRNAs).^14^ Exosomes are formed by the inward budding of multivesicular endosomes, then are fused with the plasma membrane, and released into the extracellular space to influence the function and physiology of recipient cells.^15^ They are therefore the key crosstalk factors in intercellular communication.^16^

It is well‐known that inflammatory responses are essential for heart healing after MI,^17^ while excessive inflammation could induce adverse pathological remodelling.^18^ An increasing number of studies showed that macrophages play important roles in post‐infarction inflammation and heart repair.^19^ Upon their different phenotypic and functional plasticities, macrophages are divided into two categories: M1‐like phenotype macrophages (classically activated macrophages) with high expression of CD11b^20^, ^21^ and iNOS,^22^ and elaborate large amounts of pro‐inflammatory cytokine, such as interleukin 6 (IL‐6), interleukin 1β (IL‐1β) and tumour necrosis factor‐α (TNF‐α); the M2‐like phenotype macrophages (alternatively activated macrophages) are characterized by CD206,^23^ arginase I^24^ and anti‐inflammatory cytokines (interleukin 10, IL‐10).

During the early stage of post‐infarction, M1 macrophages are recruited into infarct myocardium, showing a strong phagocytic activity; in the later phage of MI, M2 macrophages are dominant and responsible for inflammation resolution and heart healing.^25^ Recent studies have indicated that the administration of MSCs and their derived exosomes could convert the pro‐inflammatory M1 to anti‐inflammatory M2 macrophages and promote cardiac repair in MI as well.^26^, ^27^ As macrophage polarization plays an important role after MI, thereby regulating a balanced relationship between M1 and M2 macrophages might be a potentially effective therapeutic target in heart repair.

Importantly, MSCs possess some characteristics of immune‐inflammatory cells, and when treated with low concentration of pro‐inflammatory factors, such as lipopolysaccharide (LPS) or TNF‐α, MSCs remain information from danger signals and have better therapeutic efficacy in decreasing inflammation.^28^, ^29^ LPS pre‐conditioning can protect bone marrow‐derived MSCs (BMSCs) from oxidative stress‐induced apoptosis and improve the survival of BMSCs,^30^ and transplantation of LPS pre‐conditioning of BMSCs resulted in superior therapeutic neovascularization and recovery of cardiac function in a rat model of acute myocardial infarction.^31^ Recently, previous studies have indicated that LPS pre‐conditioning of BMSCs and their secreted exosomes promotes macrophage polarization and tissue repair in many inflammation diseases^32^, ^33^; however, it is incompletely elucidated in MI. The present study investigated whether LPS‐primed BMSC‐derived exosomes could mediate inflammation and myocardial injury via macrophage polarization after MI.

## 2 METHODS

### 2.1 Primary isolation, culture and identification of rat BMSCs

Bone marrow‐derived mesenchymal stem cells were isolated and identified according to previously established methods.^34^ 3~4‐week‐old male Sprague Dawley rats were used for BMSC isolation. And then, BMSCs were cultured with Dulbecco's modified Eagle's medium/nutrient mixture F‐12 (DMEM/F12), containing 10% foetal bovine serum (FBS; Biological Industries) with 100 U/mL streptomycin‐penicillin (Gibco). Bone marrow‐derived mesenchymal stem cells at passages 3‐4 were used for further experiments.

The identification of BMSCs was carried out by flow cytometry analysis with cell surface markers, including positive cluster of differentiated CD29, CD90, CD44 and negative CD45, which is described in the following.

### 2.2 Isolation, purification and identification of BMSC‐derived exosomes

When cell confluency reached 80%‐90%, the medium was removed, and the cells were washed twice with phosphate‐buffered saline (PBS) and replaced with fresh culture medium contained LPS with 10% exosome‐depleted FBS. The culture medium without LPS was used for negative control. The culturing was followed for another 24 hours, and conditioned culture medium was collected. BMSC‐derived exosomes (Exo) and LPS‐primed BMSC‐derived exosomes (L‐Exo) were isolated by density‐gradient ultracentrifugation as described previously.^35^

Nanoparticle tracking analysis (NTA) was applied to record the concentration and size distribution of exosomes using NanoSight NS300. Transmission electron microscopy (TEM) (Hitachi H7650 TEM) was used to describe the morphology of exosomes. And exosomal protein markers (eg CD63, CD81, HSP70 and Tsg101) were detected by Western blot analysis.

### 2.3 Raw264.7 cells were co‐cultured with Exo and L‐Exo

Raw264.7 (murine monocyte/macrophage) cells were purchased from American Type Culture Collection and maintained in DMEM (Gibco), containing 10% FBS with 100 U/mL streptomycin‐penicillin, and co‐cultured with different concentrations of exosome suspension (5, 10 and 20 μg/mL) as previously described.^32^, ^33^ After incubation for 24 hours, they cultured with LPS (100 ng/mL) for another 24 hours. After treatment, cells, miRNAs and medium supernatants were collected for the subsequent analysis.

### 2.4 Isolation of rat peritoneal macrophages and co‐culture with Exo and L‐Exo

In this study, 3~4‐week‐old male Sprague Dawley rats, weighing about 80‐100 g, were used for supplying peritoneal macrophages according to a method previously described.^7^ Cold PBS, containing 10% FBS, was injected into the rat peritoneal cavity. After gently massaging for 5 minutes, rat abdomen was scissored in the middle and peritoneal fluid was collected as much as possible in sterile condition. The collected fluid was centrifuged at 150 *g* for 5 minutes at 4°C to obtain the pellet, and the precipitates were re‐suspended and cultured in a 5% CO_2_‐humidified incubator. Unadherent cells were discarded by replacing the medium, and the adherent cells were peritoneal macrophages and used for co‐culturing with Exo and L‐Exo.

### 2.5 Western blot analysis

Western blot analysis was performed as previously described.^36^ In brief, the cells were collected, normalized and separated by 12% sodium dodecyl sulphate‐polyacrylamide gel electrophoresis (SDS‐PAGE) and transferred to polyvinylidene difluoride (PVDF) membranes (EMD Millipore). After 1 hour of blocking with 5% non‐fat milk in TBST at room temperature, the membranes were incubated with primary antibody (1:1000) overnight at 4°C and then were incubated with secondary antibody (1:5000) for 1 hour at room temperature. Eventually, the membranes were washed three times with TBST and detected by enhanced chemiluminescence (ECL) kit (Santa Cruz Biotechnology Inc).

### 2.6 Quantitative reverse transcription real‐time polymerase chain reaction (RT‐qPCR)

Total mRNA was isolated from the cells and mouse heart tissue using TRIzol reagent (Invitrogen). The cDNA was reverse‐transcribed using a cDNA Synthesis Kit (Takara). Then, RT‐qPCR was carried out with SYBR qPCR Mix Kit (Takara) and analysed by LightCycler 480 II (Roche). The primer sequences of mRNAs are listed in Table S1, and the relative expression levels of mRNAs were determined according to the 2^−∆∆Ct^ method.

### 2.7 Cell counting kit‐8 (CCK‐8) assay

Cell viability was detected using CCK‐8 assay (Dojindo Laboratories Co., Ltd.) according to the manufacturer's instructions. Briefly, cells were seeded in 96‐well plates; after treatment, 10 μL of CCK‐8 solution was added to each well and incubated for 1‐4 hours. Then, the absorbance was measured at 450 nm by using a microtitre plate reader.

### 2.8 Enzyme‐linked immunosorbent assay (ELISA)

Cell medium supernatants and mice serum were collected and stored at −20°C for the subsequent analysis of cytokines. ELISA kits were used as follows: rat IL‐6 (DKW130602), rat TNF‐α (DKW1317202), mouse IL‐6 (DKW12‐2060‐096), mouse TNF‐α (DKW12‐2720‐096), mouse IL‐1β (BMS6002; eBioscience) and mouse IL‐10 (BMS614‐2; eBioscience). The experiment was strictly undertaken in accordance with the manufacturer's instructions.

### 2.9 Flow cytometry analysis

When the confluency reached 80%‐90%, adherent cells (within 1 × 10^6^ cells) were lightly trypsinized and collected into the centrifuge tube, and then incubated with primary antibodies for 20 minutes at room temperature, and protected against light as well. The following monoclonal antibodies used for BMSCs were as follows: FITC‐labelled anti‐CD29, FITC‐labelled anti‐CD44, PE‐labelled anti‐CD90 and V450‐labelled anti‐CD45 (BD Biosciences). In the same manner, we used the following antibodies for macrophages: PE‐labelled anti‐CD11b (BD Biosciences) and FITC‐labelled anti‐CD206 (BioLegend). After incubation, cells were washed twice with PBS and then analysed using a FACSVerse flow cytometer (BD Biosciences). Cell apoptosis was detected using Annexin V‐FITC Apoptosis Detection Kit (BD Biosciences) as previously reported.^37^

### 2.10 Immunofluorescence

The immunofluorescence cytochemistry assay was used to detect the surface protein of macrophages as previously described.^38^ In brief, Raw264.7 cells were seeded in 24‐well plates and fixed with 4% paraformaldehyde for 30 minutes, followed by 0.05% Triton X‐100 for 10 minutes. After blocking with 1% BSA for 1 hour, anti‐CD206 (1:200) and CD11b (1:100) primary antibodies were incubated overnight at 4°C and then followed by incubation with fluorescence‐labelled goat anti‐mouse IgG (Alexa Fluor 647; 1:2000; Abcam). Following, nucleus was stained with DAPI (4′,6‐diamidino‐2‐phenylindole). At last, the fluorescence of CD206 and CD11b was detected under a confocal laser scanning microscopy (CLSM) (LSM 510 META; Carl Zeiss).

### 2.11 Induction of mice myocardial infarction and intramyocardial injection of exosomes

Mice myocardial infarction (MI) model was inducted as previously described.^39^ Here, male C57BL/6 mice, weighing between 20‐25 g, were anaesthetized with spontaneous isoflurane inhalation and intubated via oral cavity using a rodent ventilator (ALC Bio Innovations Inc). The heart was exposed between the fourth and fifth ribs by left‐side thoracotomy. Then, cardiac pericardium was incised and the left anterior descending artery (LAD) was permanently ligated using 9‐0 polyester suture. Successful coronary occlusion was confirmed by blanching of the left ventricular muscle and ST elevation on electrocardiograms to the coronary ligation.

The mice were divided into four groups according to the random principles: sham‐operated group, PBS group, Exo group and L‐Exo group. Immediately after LAD ligation, exosomes suspended in PBS were injected into myocardium at four sites around the infarct border zone.^11^ Mice were then killed at 3 and 7 days after MI for tissue harvesting to evaluate the effect of L‐Exo on post‐infarction inflammation and cardiac repair. All animal studies were undertaken in accordance with the regulations and guidelines of Institutional Animal Care and Use Committee of Guangzhou Medical University (Guangzhou, China).

### 2.12 Haematoxylin and eosin (H&E) staining and immunohistochemistry

Mice from each group were killed at 3 and 7 days after myocardial injection. The fresh heart was fixed in 4% paraformaldehyde, embedded in paraffin and sectioned at 5‐μm intervals. Then, H&E staining, immunohistochemistry staining and immunofluorescence co‐staining of heart sections were performed as described previously.^40^

### 2.13 Terminal deoxynucleotidyl transferase dUTP nick‐end labelling (TUNEL) assay

Here, 7 days after induction of myocardial infarction and myocardial injection, apoptotic cardiomyocytes in the infarcted heart were detected by a TUNEL Assay Kit (Roche) according to the manufacturer's instructions and detected by CLSM.

### 2.14 Statistical analysis

The SPSS 13.0 software (SPSS Inc) was used for statistical analysis. All data in our study were expressed as mean ± standard deviation (SD). Differences between all pairs of groups were determined by *t* test or one‐way analysis of variance (ANOVA). *P*‐value < .05 was considered statistically significant.

## 3 RESULTS

### 3.1 Characterization of LPS‐primed BMSCs and L‐Exo

Firstly, BMSCs displayed a spindle‐shaped morphology under microscopic observations (Figure S1A) and were positive for CD29, CD44 and CD90 and negative for CD45 by flow cytometry analysis (Figure S1B). Next, BMSCs were treated with various doses of LPS for different times. We observed that LPS treatment dose‐ and time‐dependently increased the expression of IL‐6 and TNF‐α at both mRNA and protein levels (Figure S1C, D), indicating the induction of inflammatory response. Moreover, CCK‐8 assay and Annexin V‐FITC/PI dual staining indicated that there was no significant induction of apoptosis occurred in BMSCs exposing to different concentrations of LPS (Figure S1E, F). Finally, low concentration of LPS (100 ng/mL) was used to stimulate BMSCs for 24 hours, and conditioned culture medium was collected and prepared for isolation of exosomes.

Exosomes were isolated by density‐gradient ultracentrifugation (Figure S2). The exosomes showed a cup‐shaped morphology by TEM, and the size of exosomes was about 100 nm by NTA (Figure S3A, B). Exosomal markers, such as CD63, CD81, TSG101 and Hsp70, were highly expressed in both Exo and L‐Exo groups (Figure S3C). After evaluating the concentration of Exo and L‐Exo, we found that the amount of L‐Exo (1.15 ± 0.05 × 10^9^ particles/1 × 10^6^ cells) was approximately 1.2 times of Exo (0.91 ± 0.12 × 10^9^ particles/1 × 10^6^ cells) (*P* < .05) (Figure S3D). However, there was no significant difference in protein concentration between Exo (9.2 μg/1 × 10^9^ particles) and L‐Exo (9.9 μg/1 × 10^9^ particles) (Figure S3E).

### 3.2 L‐Exo attenuated inflammation by increasing anti‐inflammatory cytokines, as well as decreasing pro‐inflammatory cytokines in Raw264.7 cells

**Figure 1:**
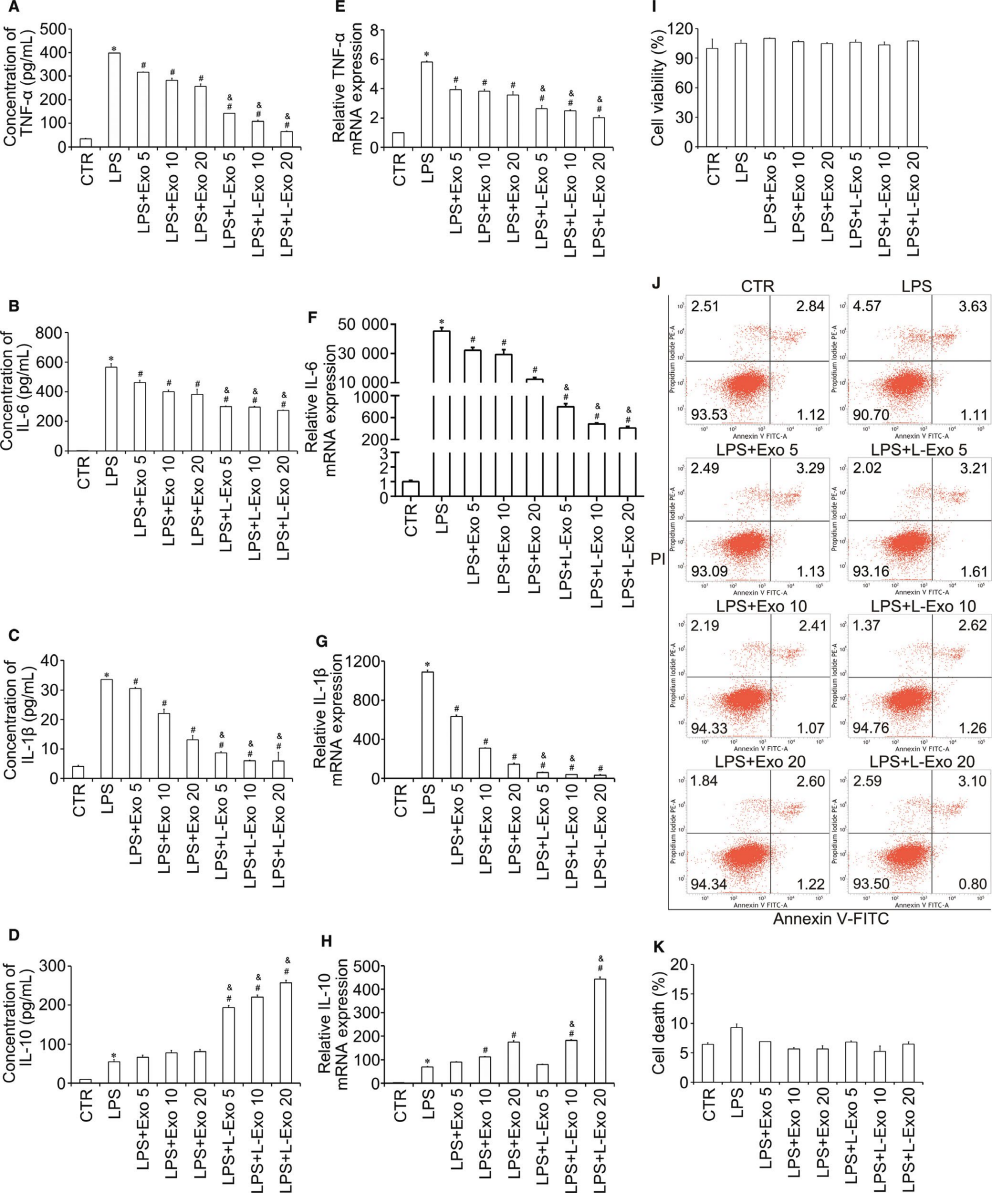
The co‐culture of Raw264.7 cells with Exo and L‐Exo by an exosomal dose‐dependent manner under LPS stimulation. After co‐culture with different doses of Exo and L‐Exo (5, 10, and 20 μg/mL) for 24 h, Raw264.7 cells were treated with LPS (100 ng/mL) for another 24 h. Followed by treatments, Raw264.7 cells and medium supernatants were collected. The secretions of pro‐inflammatory cytokines (IL‐6, TNF‐α and IL‐1β) and anti‐inflammatory cytokine (IL‐10) derived from Raw264.7 cells were detected by ELISA (A‐D), and the gene expression of TNF‐α, IL‐6, IL‐10 and IL‐1β in Raw264.7 cells was performed by RT‐qPCR (E‐H). The influences of Exo and L‐Exo on cell viability and death were detected by CCK‐8 (I) and flow cytometry analysis (J, K). *P < .05 vs CTR group, ^#^ *P* < .05 vs LPS group, ^&^ *P* < .05 vs Exo group under equal concentration

To understand the effect of Exo and L‐Exo on inflammation, we co‐cultured Raw264.7 cells with Exo and L‐Exo under LPS stimulation. Firstly, after co‐culturing Raw264.7 cells with Exo and L‐Exo at dose of 10 ug/mL for 0, 3, 6, 12 and 24 hours in advance before treated with LPS (100 ng/mL) for 24 hours, we found that pre‐incubation with Exo and L‐Exo attenuated inflammation in a time‐dependent manner and achieved significant effect at 24 hours (Figure S4A, B). Secondly, to optimize the reasonable dose of exosomes to be co‐cultured, we evaluated the effects of different doses of exosomes (5, 10 and 20 μg/mL) on inflammatory response. ELISA showed that the pro‐inflammatory cytokines (IL‐6, TNF‐α and IL‐1β) were decreased and the anti‐inflammatory cytokine (IL‐10) was increased in both Exo and L‐Exo groups, by an exosomal dose‐dependent manner compared with LPS group (*P* < .05) (Figure 1A‐D). And RT‐qPCR showed the same results (*P* < .05) (Figure 1E‐H). Compared with Exo group, we found that L‐Exo could further significantly reduce inflammation in the condition of equal exosomal dose (*P* < .05). Lastly, we selected the middle dose (10 μg/mL) of exosomes to be co‐cultured with Raw264.7 under LPS (100 ng/mL) stimulation for different times (6, 12 and 24 hours). The results illustrated that the secretion and mRNA expression of TNF‐α, IL‐6 and IL‐1β were significantly affected in a time‐dependent manner, and L‐Exo had superior effects on inflammation resolution compared with Exo at 24 hours (*P* < .05) (Figure S5A‐F).

To confirm that Exo and L‐Exo reduced inflammation without affecting cell viability and apoptosis, we performed CCK‐8 assay and flow cytometry analysis, in which the results indicated that there was no significant influence on cell viability and apoptosis (Figure 1I‐K).

### 3.3 Compared with Exo, L‐Exo could extremely increase M2 macrophage polarization and decrease M1 macrophage polarization in vitro

**Figure 2:**
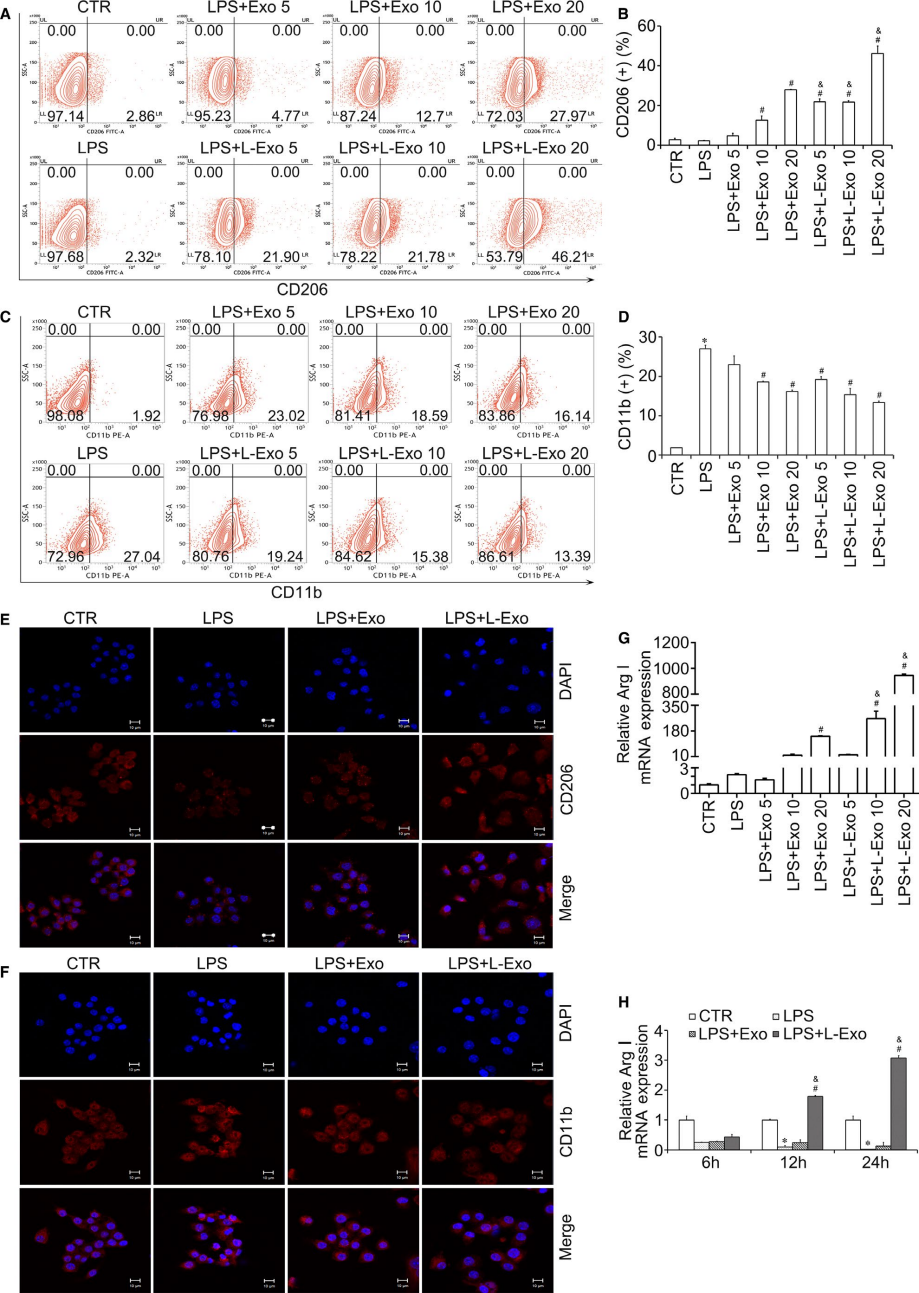
The effects of Exo and L‐Exo on macrophage polarization. The positive expression level of CD206 and CD11b in Raw264.7 cells after co‐culture with Exo and L‐Exo under LPS (100 ng/mL) stimulation was detected by flow cytometry (A, C), and the percentages (%) were accordingly calculated (B, D). The protein expression level of CD206 and CD11b was further confirmed by CLSM (E, F). M2 macrophage marker (Arg I) was measured by RT‐qPCR, according to exosomal dose‐dependent (G) and time‐dependent (H). *P < .05 vs CTR group, ^#^ *P* < .05 vs LPS group, ^&^ *P* < .05 vs Exo group under equal concentration

As described in previous studies, the inflammation is mainly associated with macrophage transition.^21^ M1 macrophages mostly elaborate pro‐inflammatory cytokines (IL‐6, TNF‐α and IL‐1β), and M2 macrophages mainly secrete anti‐inflammatory cytokine (IL‐10) (Figure 1). We distinguished macrophage subtypes by detecting protein markers (CD11b, iNOS (M1 macrophages) and CD206, ArgI (M2 macrophages)). The flow cytometry analysis showed that Exo and L‐Exo markedly reduced the positive incidence level of CD11b and significantly increased the positive incidence level of CD206, by an exosomal dose‐dependent manner compared with LPS group (*P* < .05) (Figure 2A‐D). In addition, immunofluorescence staining further confirmed that the expression level of CD206 was distinctly increased and CD11b was obviously decreased in both Exo group and L‐Exo group, and it seemed that L‐Exo had better effect according to our observations (Figure 2E, 2). Furthermore, the RT‐qPCR results showed that the M2 macrophage protein marker (ArgI) was remarkably increased by an exosomal dose‐dependent and time‐dependent manners in L‐Exo group (*P* < .05) (Figure 2G, H).

**Figure 3:**
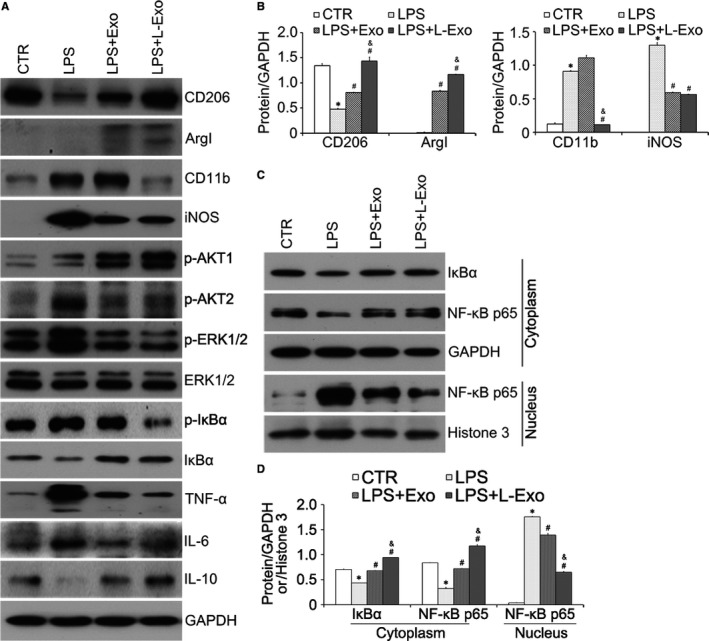
The effects of L‐Exo on inflammation and polarization of macrophage mainly by inhibiting NF‐κB signalling pathway in Raw264.7 cells. Raw264.7 cells were pre‐cultured with Exo and L‐Exo at dose of 10 μg/mL for 24 h before treatment, and with LPS (100 ng/mL) for another 24 h. (A, B) The protein amounts of CD206, CD11b, ArgI, iNOS, IL‐6, IL‐10, TNF‐α, ERK1/2, p‐ERK1/2, p‐IκBα, IκBα, p‐AKT1 and p‐AKT2 were assayed by Western blot analysis, and the relative expression of CD206, CD11b, ArgI and iNOS was normalized by GAPDH. (C, D) Cytoplasmic and nuclear proteins were collected and extracted to measure IκBα and NF‐κB p65. GAPDH and Histone3 were used as normalized cytoplasmic and nuclear protein. *P < .05 vs CTR group, ^#^ *P* < .05 vs LPS group, ^&^ *P* < .05 vs Exo group

Similarly, results of Western blot analysis demonstrated that the protein amounts of CD206, ArgI and IL‐10 were increased, while the protein amounts of CD11b, iNOS, IL‐6 and TNF‐α were decreased in L‐Exo group compared with LPS and Exo group (*P* < .05) (Figure 3A, B). The above results showed that L‐Exo had superior effects on promoting M2 macrophage polarization and decreasing M1 macrophage polarization compared with Exo under equal exosomal dose concentration (*P* < .05).

### 3.4 L‐Exo reduced the inflammation of macrophages by suppressing the NF‐κB signalling pathway and regulated the macrophage polarization partially by the AKT1/AKT2 signalling pathway

We further analysed the molecular mechanism of macrophage polarization regulated by Exo and L‐Exo. Based on elaboration of typical inflammatory factors (TNF‐α, IL‐6 and IL‐1β), we discussed the effects of Exo and L‐Exo on activation of LPS‐dependent NF‐κB signalling pathway. Western blot results showed that total protein of IκBα was increased and p‐IκBα was decreased in both Exo group and L‐Exo group after LPS treatment compared with LPS group (Figure 3A). Besides, NF‐κB p65 translocation from the cytoplasm to nucleus was significantly reduced (Figure 3C). Normalized by GAPDH and Histone3, we found L‐Exo had better effects on inhibiting nuclear translocation of NF‐κB p65 compared with Exo group (*P* < .05) (Figure 3D).

**Figure 4:**
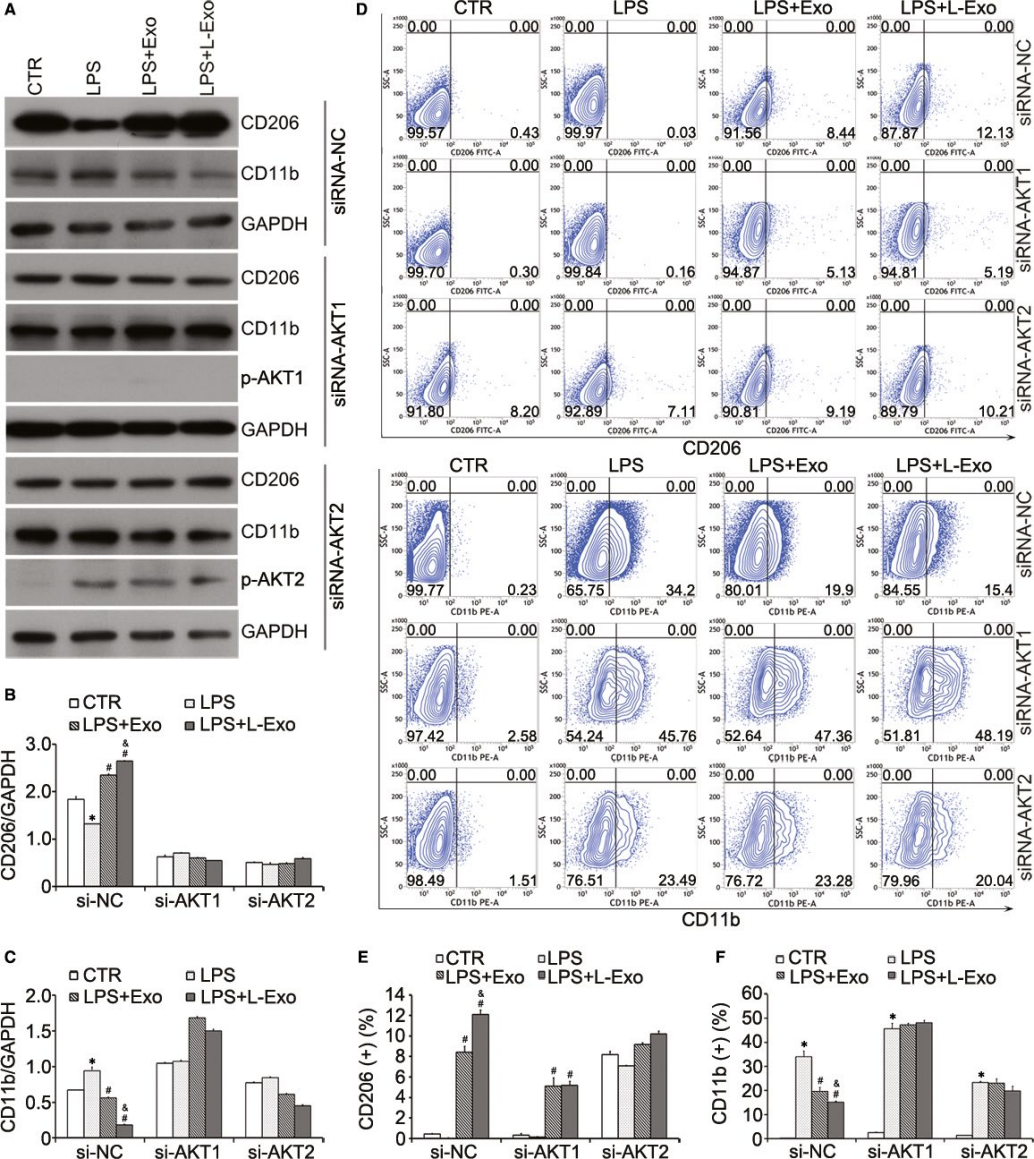
The effects of L‐Exo on macrophage polarization were regulated by AKT1/AKT2 signalling pathway in Raw264.7 cells. Raw264.7 cells were transfected with siRNA targeting AKT1 and AKT2 for 12 h before co‐culture with exosomes and treatment by LPS. (A‐C) The expression level of macrophage protein markers (CD206 and CD11b) was assayed by Western blot analysis and normalized by GAPDH. (D‐F) The representative flow cytometry plots and positive percentages of CD206 and CD11b were shown. *P < .05 vs CTR group, ^#^ *P* < .05 vs LPS group, ^&^ *P* < .05 vs Exo group

In this study, we found that phosphorylation activation of AKT1 was excited in M2 macrophages, while phosphorylation activation of AKT2 was increased in M1 macrophages by Western blot analysis (Figure 3A). Then, we transfected Raw264.7 cells with siRNA targeting AKT1 and AKT2. Western blot analysis showed that knockdown of AKT1 and AKT2 diminished the effects of Exo and L‐Exo groups on macrophage polarization (Figure 4A‐C). Similarly, the results of flow cytometry analysis showed that silencing AKT1 reduced the positive incidence level of CD206 and significantly increased the positive incidence level of CD11b while silencing AKT2 partially reversed the above outcome (Figure 4D‐F).

### 3.5 L‐Exo reduced the inflammation of rat primary peritoneal macrophages (Mφ) and promoted M2 macrophage polarization

**Figure 5:**
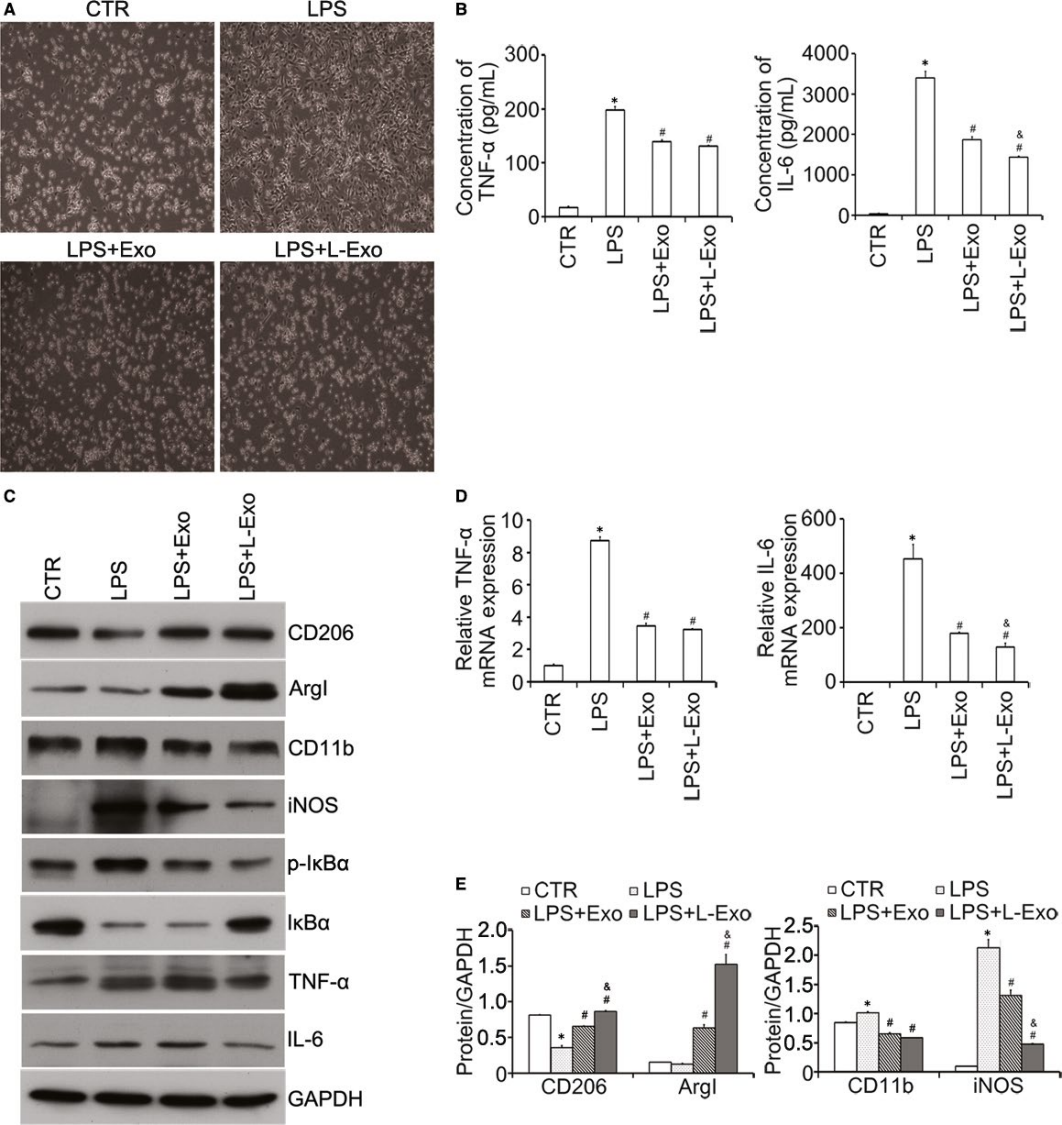
The effects of L‐Exo on inflammation and phenotype of rat peritoneal macrophages under LPS treatment. Firstly, rat peritoneal macrophages were pre‐cultured with Exo and L‐Exo at dose of 10 μg/mL for 24 h and then followed by treatment with LPS (500 ng/mL) for 24 h. A, The characteristic morphology of rat peritoneal macrophages co‐cultured with Exo and L‐Exo under LPS stimulation was shown (200×). B, C, The cytokine secretions and gene expression of TNF‐α and IL‐6 were measured by ELISA. D, E, Total protein amounts of CD206, CD11b, ArgI, iNOS, p‐IκBα, IκBα, IL‐6 and TNF‐α were determined by Western blot analysis and normalized by GAPDH. *P < .05 vs CTR group, ^#^ *P* < .05 vs LPS group, ^&^ *P* < .05 vs Exo group

To further validate the effects of Exo and L‐Exo on transition of macrophages in vitro, we co‐cultured Exo and L‐Exo with rat primary peritoneal macrophages (Mφ). Firstly, the morphology of Mφ in Exo and L‐Exo groups maintained oval shape, which were different from LPS group, characterized with triangular shape and tentacles (Figure 5A). Additionally, the production of TNF‐α and IL‐6 from Mφ in Exo group and L‐Exo group was significantly decreased by ELISA and RT‐qPCR compared with LPS group (*P* < .05), and L‐Exo exhibited more beneficial function in reducing inflammation compared with Exo (*P* < .05) (Figure 5B, C). Moreover, the protein amounts of CD206 and ArgI were increased and those for CD11b and iNOS were decreased in both Exo group and L‐Exo group (Figure 5D). After normalized by GAPDH, the results were superior in L‐Exo group (*P* < .05) (Figure 5E). These findings suggested that, compared with Exo, L‐Exo could further promote M2Mφ polarization and attenuate M1Mφ polarization under LPS stimulation.

### 3.6 Compared with Exo, L‐Exo preferably reduced post‐infarction inflammation and relieved myocardial injury after myocardial infarction

**Figure 6:**
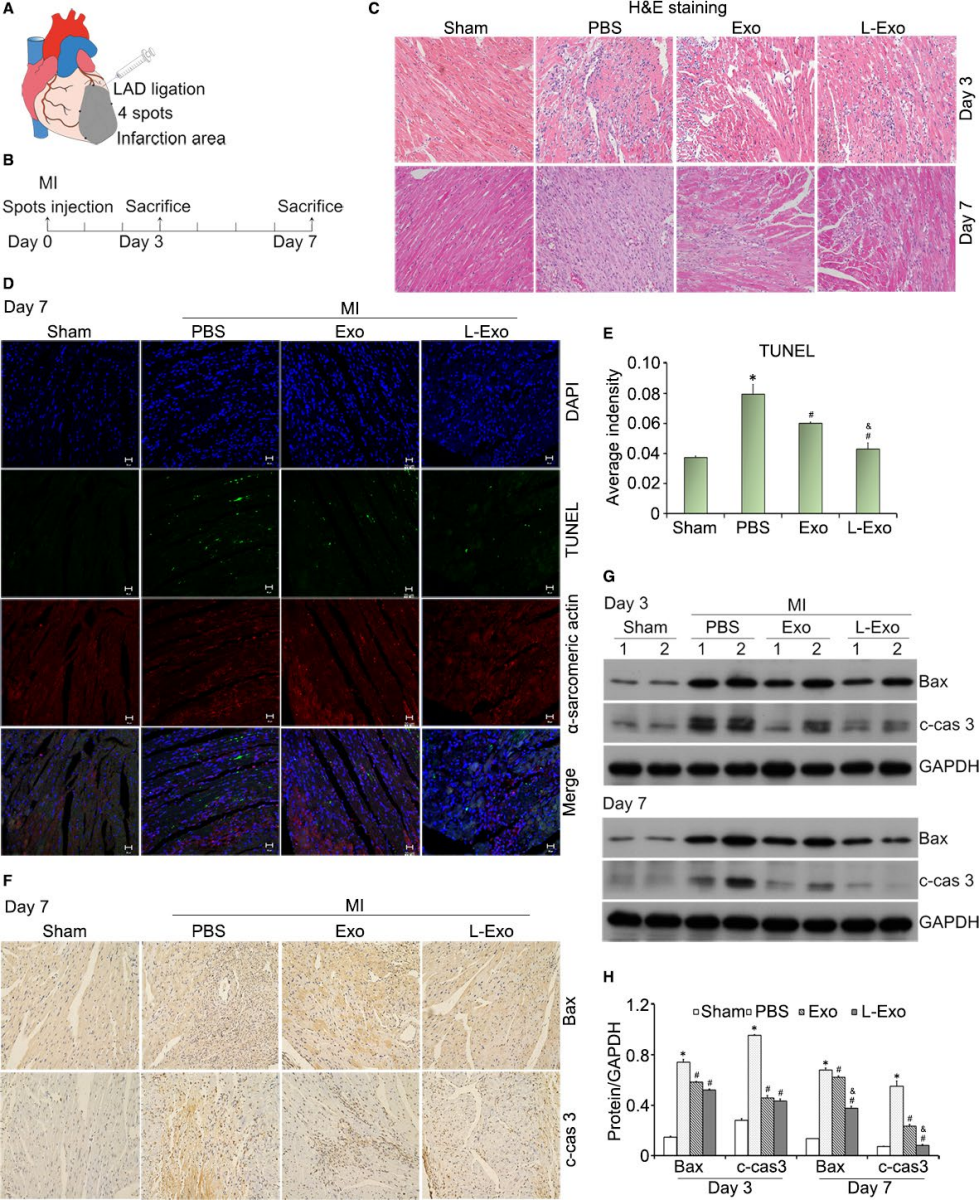
Intramyocardial injection of L‐Exo attenuated cardiomyocyte apoptosis in MI mice. A, The diagrammatic sketch of mice myocardial infarction induction and exosome intramyocardial injection. B, Mice in each group were killed at 3 and 7 d after intramyocardial injection (n ≥ 12). C, Representative images of inflammatory cells infiltration within the infarct border zone at 3 and 7 d were illustrated by H&E staining. D, The apoptosis of cardiomyocytes at 7 d in the ischaemic myocardium was assessed by a TUNEL Assay Kit. Nucleus was blue with DAPI, α‐sarcomeric actin (SA)‐positive cardiomyocytes were red, and the TUNEL‐positive apoptotic cardiomyocytes were green under CLSM. E, The average fluorescence intensity of positive apoptotic cardiomyocytes was quantified. F, The protein of Bax and cleaved caspase‐3 within the ischaemic heart tissue at 7 d was analysed by immunohistochemical staining (200×, respectively). G, H, Western blotting validated the protein expression of Bax and cleaved caspase‐3 in ischaemic heart tissue at 3 and 7 d post‐MI, normalized by GAPDH. *P < .05 vs sham group, ^#^ *P* < .05 vs PBS group, ^&^ *P* < .05 vs Exo group

After successful LAD ligation, mice were submitted to exosome treatment and 50 μg of Exo or L‐Exo in 50 μL PBS was injected into the infarct border zone at four spots (Figure 6A). Then, mice were killed at 3 and 7 days for tissue harvesting (Figure 6B).

The induction of myocardial infarction induced post‐infarction inflammation, and a large number of inflammatory cells assembled and infiltrated into the infarction area after MI. H&E staining showed that the administration of Exo and L‐Exo could alleviate the infiltration of inflammatory cells to protect myocardium from further damage (Figure 6C). After MI, cardiomyocyte apoptosis occurred along with ischaemia and hypoxia, as well as myocardium inflammation. TUNEL assay was used to detect the cardiomyocyte apoptosis. Under CLSM, TUNEL‐positive (green) apoptotic cardiomyocytes (α‐sarcomeric actin, red) in Exo group and L‐Exo group were decreased compared with PBS group (Figure 6D). After quantification of the average fluorescence intensity, L‐Exo group exhibited better effects compared to other groups (*P* < .05) (Figure 6E). Immunohistochemical staining along with Western blot analysis further confirmed the cardioprotective effect of Exo and L‐Exo with reducing protein amounts of Bax and cleaved caspase‐3 in ischaemic heart tissue (Figure 6F, G). Normalized by GAPDH, we found that the apoptosis of cardiomyocytes in L‐Exo group was significantly reduced compared with Exo group and PBS group (*P* < .05) (Figure 6H).

**Figure 7:**
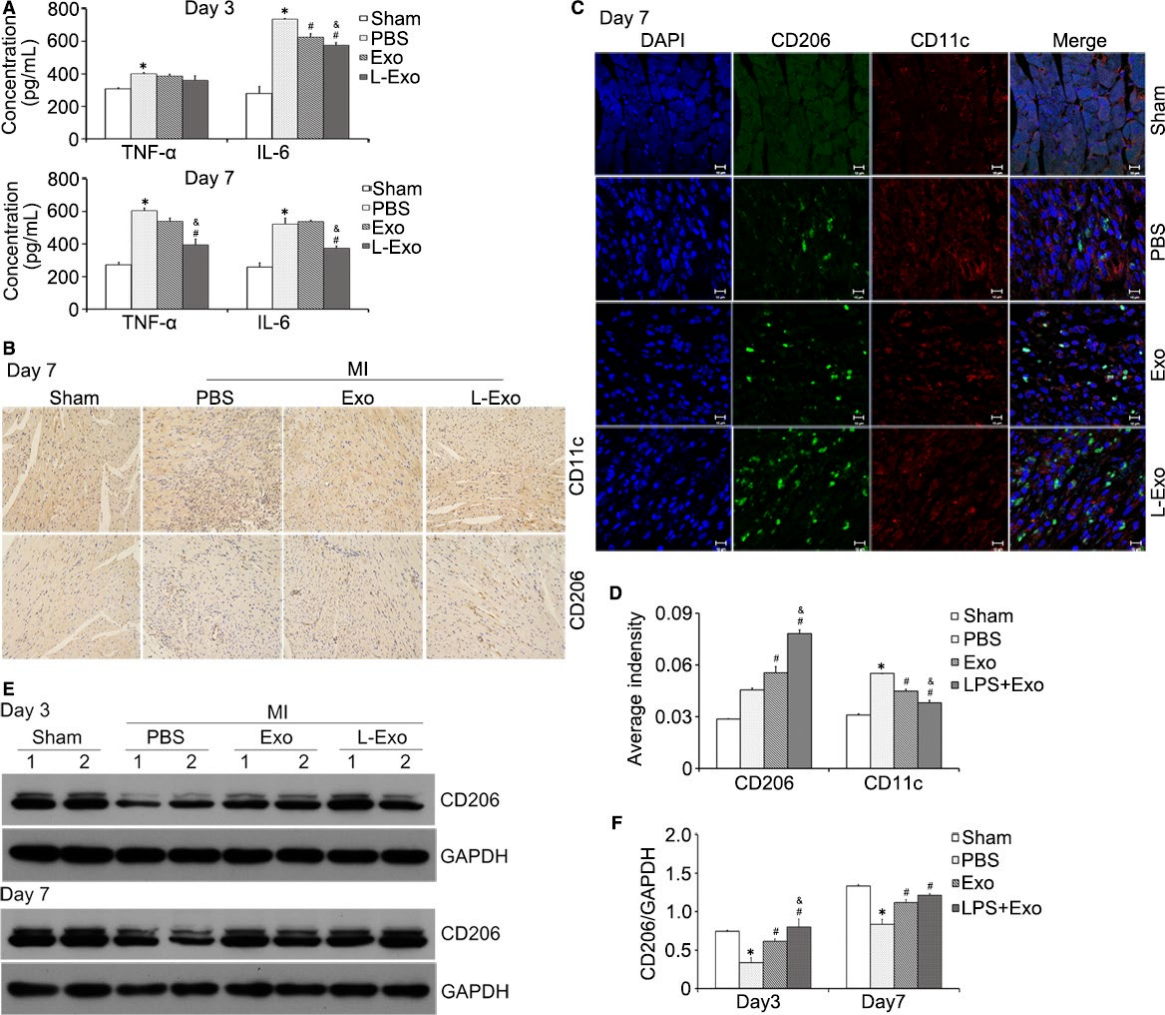
L‐Exo administration reduced post‐infarction inflammation by increasing M2 macrophage polarization in mice. A, Serum inflammatory cytokines (TNF‐α and IL‐6) were assayed by ELISA. B, Immunohistochemical staining of CD206 (M2 marker) and CD11c (M1 marker) at 7 d (200×, respectively). C, Representative images of immunofluorescence co‐staining. DAPI (blue), CD11c (red) and CD206 (green). D, The average fluorescence intensity of CD11c and CD206 was quantified. E, F, Representative images of Western blot for CD206 in ischaemic heart tissue at 3 and 7 d post‐MI, normalized by GAPDH. *P < .05 vs sham group, ^#^ *P* < .05 vs PBS group, ^&^ *P* < .05 vs Exo group

After MI, post‐infarction inflammation induced a great amount of cytokines elaborating into the blood circulation. We detected the pro‐inflammatory cytokines (TNF‐α and IL‐6) in serum at 3 and 7 days. Compared with Exo group, the secretion of TNF‐α and IL‐6 in serum was significantly reduced in L‐Exo group (*P* < .05) (Figure 7A).

Additionally, we analysed the subsets of macrophages gathering into the ischaemic heart. Immunohistochemical staining showed that the protein expression level of CD206 (M2 marker) was higher in both Exo group and L‐Exo group while CD11c (M1 marker)^41^, ^42^ was lower (Figure 7B). Immunofluorescence co‐staining demonstrated, after quantifying the relative fluorescence intensity of CD11c and CD206, we discovered that L‐Exo effectively increased the expression of CD206 and decreased the expression of CD11c compared with Exo group and PBS group (Figure 7C, D). Western blot analysis further verified that the total protein amount of CD206 in ischaemic heart tissue was elevated with treatment of Exo and L‐Exo (Figure 7E). Normalized by GAPDH, L‐Exo presented better effects than Exo on facilitating M2 macrophage polarization after MI in mice (Figure 7F).

Taken together, compared with Exo, the administration of L‐Exo could attenuate cardiomyocyte apoptosis and relieve myocardial injury after MI. Furthermore, L‐Exo could reduce post‐infarction inflammation with promoting M2 macrophage and degrading M1 macrophage polarization in mice.

## 4 DISCUSSION

Recently, exosomes serve as substitutes for BMSCs in cell‐free therapy, playing important roles in mediating heart repair during myocardial infarction injury.^11^, ^43^ The mechanisms of cardioprotection of BMSC‐derived exosomes have not been fully elucidated, while the effects of anti‐inflammation, immunoregulation, angiogenesis and the modulation of M1/M2 macrophage polarization have gradually attracted scholars’ attention.^44^ Macrophages are involved in heart injury and repair after MI and can be switched from one subset to another based on different microenvironments.^25^ Recent studies have proved that LPS‐pre‐conditioned MSC‐derived exosomes (LPS pre‐Exo) greatly alleviated inflammation in THP‐1 cells and enhanced diabetic cutaneous wound healing mainly by promoting M2 macrophage activation,^32^ but whether LPS pre‐Exo could improve heart repair via modulation of macrophage phenotype has not been fully elucidated in MI. In the current study, we found that exosomes derived from BMSCs, both Exo and L‐Exo, could transform macrophage subset from M1 phenotype to M2 phenotype in vitro under LPS stimulation, and reduced post‐infarction inflammation with promoting M2 macrophage and degrading M1 macrophage polarization in ischaemic heart. What caught our attention was that L‐Exo exhibited superior therapeutic effects than Exo.

The specific molecular mechanism of macrophage polarization has not been clearly acknowledged. Numerous studies have revealed that the activation and polarization of macrophages could be mediated by many signalling pathways, including PI3K/AKT, JAK/STAT and NF‐κB signalling pathways.^45^, ^46^ In LPS‐induced inflammation, the NF‐κB signalling pathway is the most important activation and effector pathway, in which IκB is an inhibitor of NF‐κB, and the activation of IκB promotes NF‐κB p65 to translocate to the cell nucleus and induces transcription and secretion of pro‐inflammatory cytokines.^47^ In our study, firstly we observed that L‐Exo significantly reduced the phosphorylation level of IκB, which indicated that L‐Exo might inhibit the LPS‐dependent NF‐κB signalling pathway. And we finally discovered that L‐Exo preferably inhibited the translocation of NF‐κB p65 from cytoplasm to nucleus in Raw264.7 cells under LPS stimulation, reducing the elaboration of pro‐inflammatory cytokine of IL‐6, TNF‐α and IL‐1β.

In addition, the NF‐κB signalling pathway is the terminal link of LPS‐induced inflammation, and its upstream may be regulated by a variety of signalling pathways. The AKT signalling pathway is involved in the regulation of various cellular functions and plays an important role in inflammation and macrophage polarization.^48^ Previous studies have shown that different subtypes of AKT may act as upstream regulators of NF‐κB, mediating inflammation and polarization of macrophages. Especially, AKT1 mainly mediates M2 macrophage polarization, and AKT2 mostly regulates M1 macrophages polarization.^45^ Deficiency of AKT2 promotes M2 macrophage polarization and suppresses M1 macrophage priming,^49^ while AKT1 ablation gives rise to an M1 phenotype.^50^ Therefore, we gave an insight into AKT1/AKT2 isoform activation. In our study, we found that L‐Exo could significantly activate phosphorylation of AKT1 and promote M2 macrophage polarization, while suppressing phosphorylation activation of AKT2 and reducing M1 macrophage polarization. When respectively knocking down AKT1 and AKT2, we found that the efficacy of L‐Exo on macrophage polarization was partially diminished, which indicated that L‐Exo regulated the macrophage polarization partially by the AKT1/AKT2 signalling pathway.

After MI, macrophages migrate into the ischaemic cardiac tissue and participate in inflammation and cardiac repair. Several studies have demonstrated that depletion of macrophages prevents wound healing and increases left ventricular remodelling after myocardial injury.^51^, ^52^ Moreover, depletion of macrophages impairs the cardioprotective effects of MSC‐Exo after myocardial ischaemia/reperfusion injury, suggesting that MSC‐Exo mediates heart repair largely relying on its interaction with macrophages.^53^ In our present study, the administration of Exo and L‐Exo by intramyocardial injection at infarct border zone could reduce the infiltration of inflammatory cells, attenuate inflammation and effectively transmit macrophage subsets from M1 to M2 in the ischaemic heart tissue.

The ischaemia and hypoxia, as well as myocardium inflammation, could induce cardiomyocyte apoptosis after MI. Cardiomyocyte apoptosis is the main cause of myocytes loss, which is associated with heart remodelling in several cardiovascular diseases. In the previous study, human BMSCs increased the frequency of alternatively activated anti‐inflammatory monocytes/macrophages (M2) infiltrating into injured myocardium and reduced apoptotic cardiomyocytes in the infarct area after acute MI.^27^ Increasing Evidence have shown that MSC‐derived exosome treatment could remarkably decrease cardiomyocyte apoptosis and improve cardiac function. Especially, in dilated cardiomyopathy, MSC‐derived exosomes could alleviate inflammatory cardiomyopathy and reduce cardiomyocyte apoptosis by regulating the anti‐inflammatory macrophage polarization.^54^ In this article, we discovered L‐Exo could beneficially attenuate post‐infarction inflammation and cardiomyocyte apoptosis, and significantly facilitate the polarization of M2 macrophages at the same time. What is more, we followed up for 3 months to detect the ejection fraction of left ventricle by echocardiography and found some therapeutic benefits in improving short‐term cardiac function. It suggested that L‐Exo contributed to relieving myocardial injury and ameliorating cardiac function after MI. Therefore, concerning the expand of sample size, further studies need to be conducted.

Increasing Evidence have demonstrated that pre‐conditioning could enhance the therapeutic effects of MSCs.^55^, ^56^ Compared to in vivo inflammatory licensing by the recipient environment alone, in vitro inflammatory licensing prior to clinical use could enhance the immunomodulatory ability of MSCs exposed to inflammatory macrophages and secretome from primed MSCs possessed biologically active.^57^ TLR4 activation by LPS stimulation could enhance the paracrine capacity of BMSCs, releasing large amounts of cytokines and exosomes, to represent a physiologically significant mechanism for tissue repair.^58^ In our study, we found that LPS pre‐conditioning BMSCs produced more exosomes and L‐Exo showed superior effects than Exo both in vitro and in vivo. Evidence showed that LPS‐pre‐conditioned MSC‐derived exosomes reduced inflammation and promoted wound healing through modulating the M2 macrophage activation by shuttling let‐7b.^32^ Similarly, IL‐1β‐primed human umbilical cord MSC‐derived exosomes effectively induced macrophage polarization towards an anti‐inflammatory M2 phenotype partially through the transfer of exosomal miR‐146a.^33^ In addition, LPS pre‐conditioning could activate BMSCs, producing several other factors, such as fibroblast growth factor 2 (FGF2), vascular endothelial growth factors (VEGF), hepatocyte growth factor (HGF), insulin‐like growth factor 1 (IGF‐1) and TGF‐β, which may be packed in exosomes to exert cardioprotection after MI.^31^ It is possible that some beneficial proteins and RNAs in L‐Exo may play important roles^59^; however, the underlying mechanisms have not been fully clarified yet.

Given the emerging therapeutic potential of inflammatory priming BMSC‐derived exosomes instead of BMSCs themselves in cardiovascular diseases, the effectively bioactive components contained in exosomes play important roles in interaction with recipient cells. And the cargoes in exosomes, such as proteins and RNAs, will change as responding to different microenvironments. Therefore, exosomes could serve as potential biomarkers to reflect the state of diseases and are used as a new therapeutic target for future therapy.^60^ In our following study, we will devote to understand the exosomal proteins and RNAs, respectively, and draw a new perspective into the potential regulation mechanism of L‐Exo, which participate in heart repair after MI.

In conclusion, our findings demonstrated that in contrast with Exo, L‐Exo had superior therapeutic effects on promoting M2 macrophage polarization in vitro, as well as attenuating the post‐infarction inflammation and cardiomyocyte apoptosis by mediating macrophage polarization in mice myocardial infarction model. Consequently, we have confidence in the perspective that low concentration of LPS pre‐conditioning BMSC‐derived exosomes may develop into a promising cell‐free treatment strategy for clinical treatment of MI.

## CONFLICT OF INTEREST

The authors declare that they have no conflict of interest.

## AUTHOR CONTRIBUTIONS

NL and SL designed the experiments; RX, F.ZH, RC, W.ZH., MH, B.L, XC, M.L and QX performed the experiments; and NL, SL and RX wrote the manuscript. All authors read and approved the final manuscript.

## Data Availability

The data that support the findings of this study are available from the corresponding author upon reasonable request.
