# Supplementary figures and images for: Exosomes derived from pro‐inflammatory bone marrow‐derived mesenchymal stem cells reduce inflammation and myocardial injury via mediating macrophage polarization
Source: J Cell Mol Med. 2019 Sep 26;23(11):7617–31. doi: 10.1111/jcmm.14635 (PMC6815833; doi:10.1111/jcmm.14635)

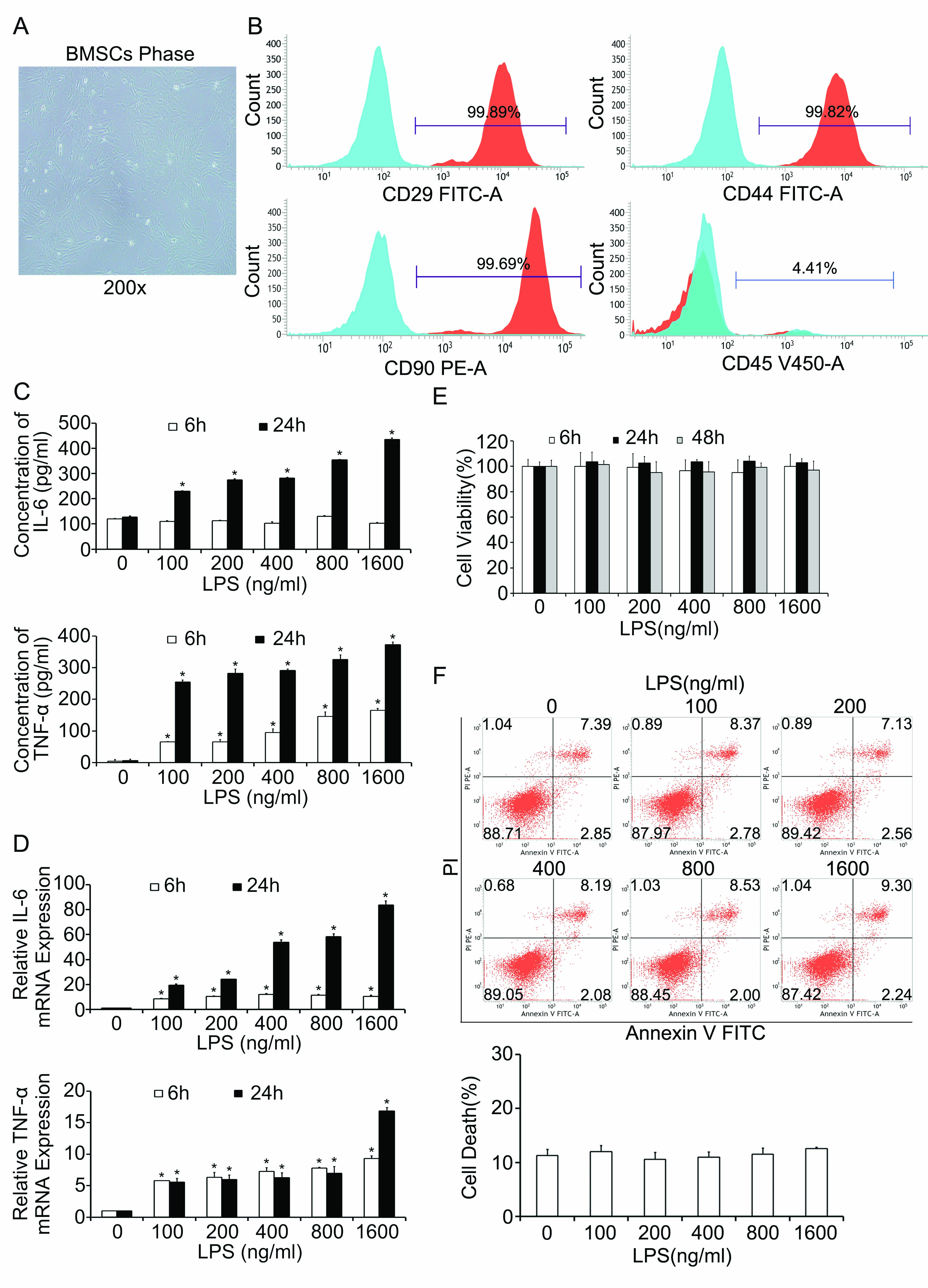

Supplement: Supplementary file 1 [file JCMM-23-7617-s001.jpg]

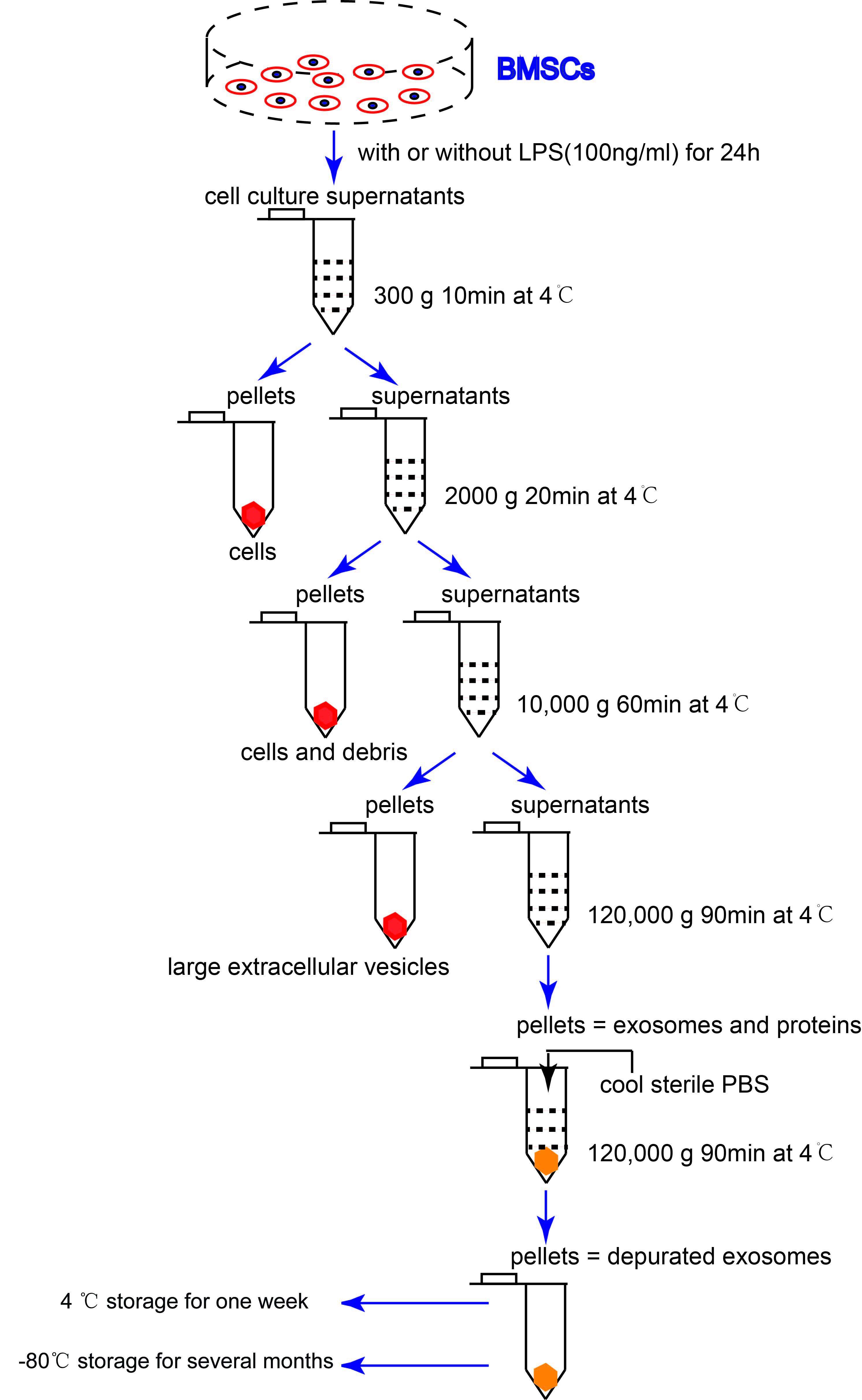

Supplement: Supplementary file 2 [file JCMM-23-7617-s002.jpg]

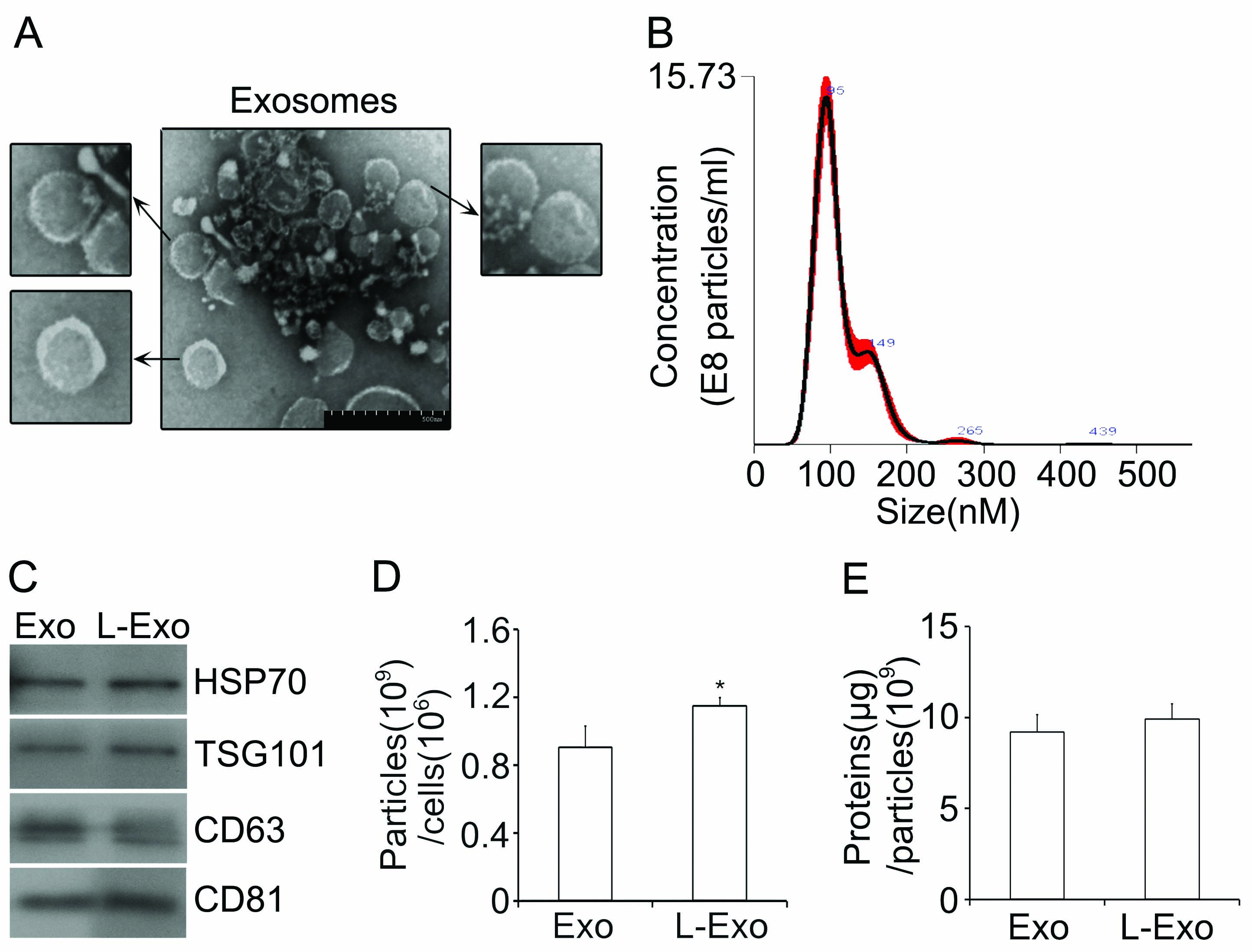

Supplement: Supplementary file 3 [file JCMM-23-7617-s003.jpg]

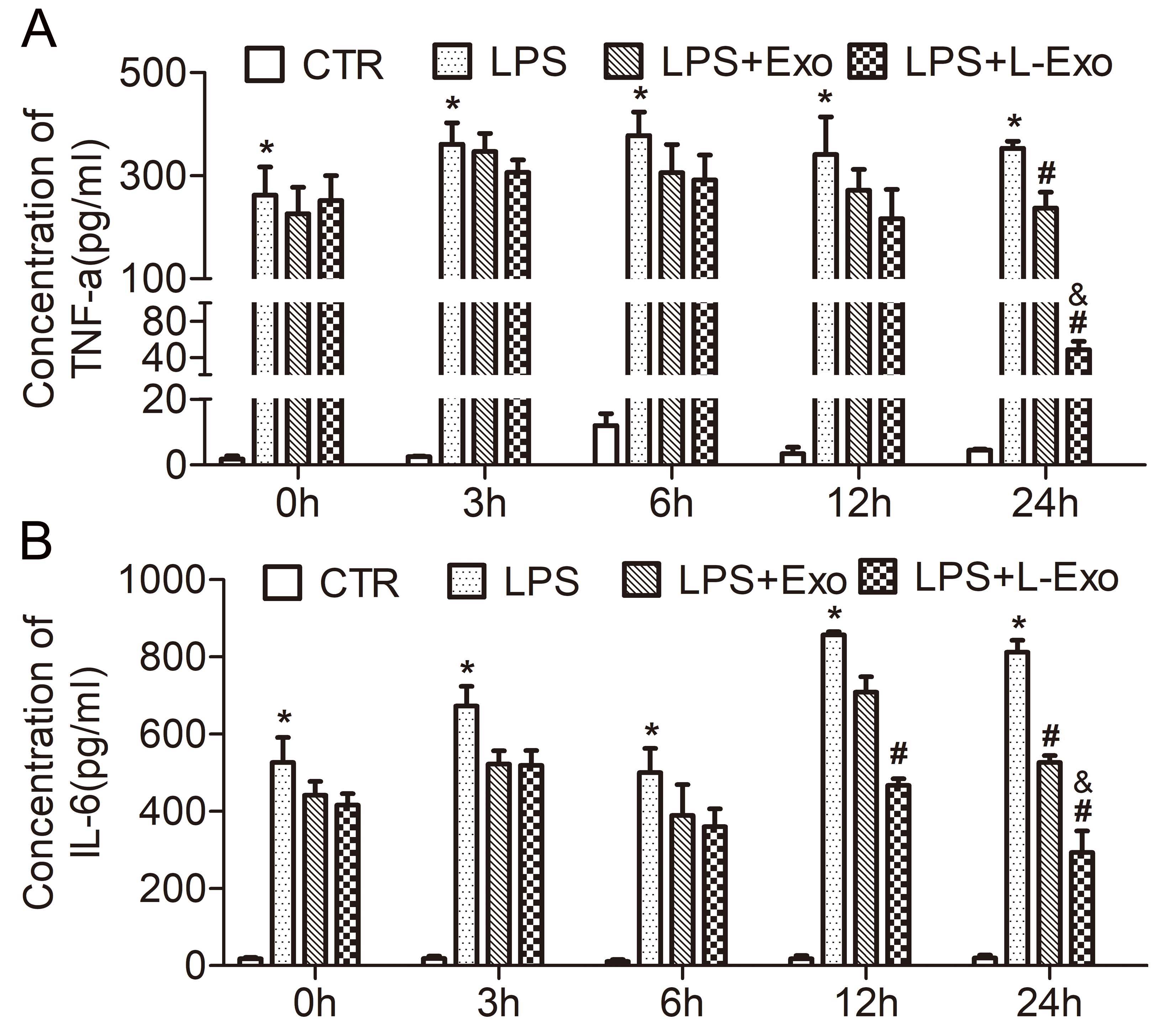

Supplement: Supplementary file 4 [file JCMM-23-7617-s004.jpg]

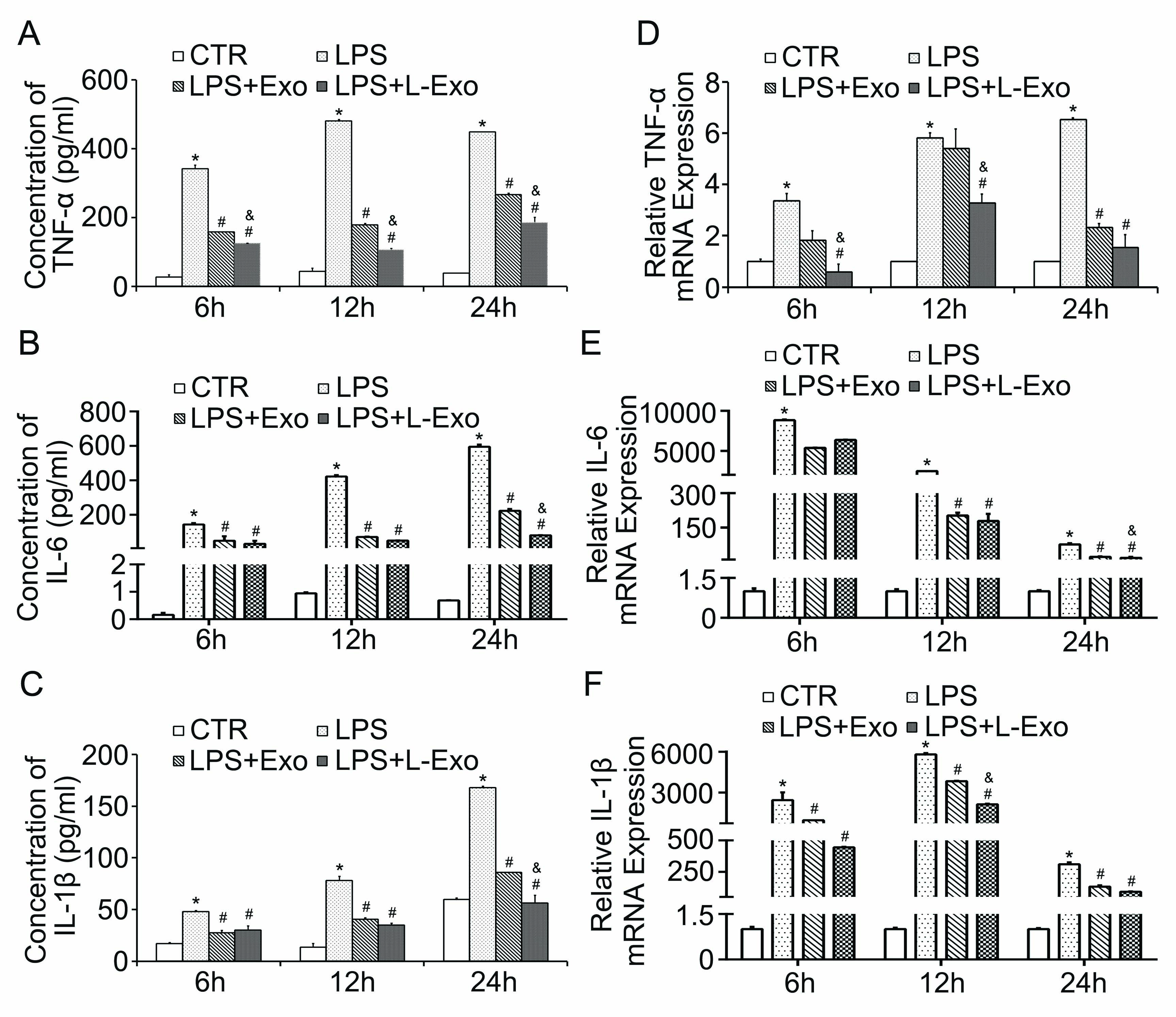

Supplement: Supplementary file 5 [file JCMM-23-7617-s005.jpg]
