# Supplementary material for: Exosomes derived from pro‐inflammatory bone marrow‐derived mesenchymal stem cells reduce inflammation and myocardial injury via mediating macrophage polarization
Source: J Cell Mol Med. 2019 Sep 26;23(11):7617–31. doi: 10.1111/jcmm.14635 (PMC6815833; doi:10.1111/jcmm.14635)
Supplement: Supplementary file 6 [file JCMM-23-7617-s006.docx]

**
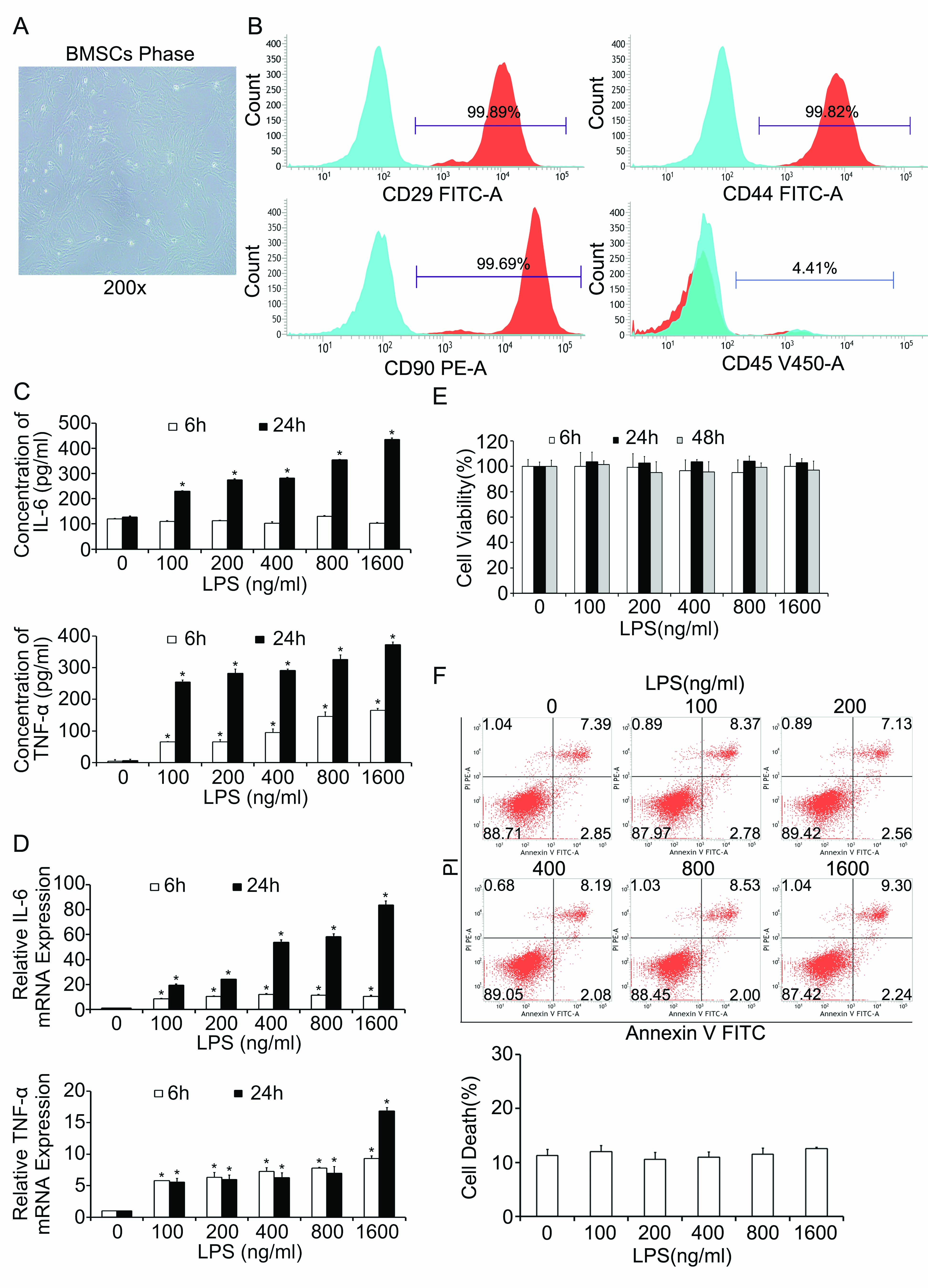
**

**Fig. S1.** **Characterization of BMSCs and preconditioning with LPS.** (A) The typical morphology of BMSCs in passage 2 under light microscope. (B) BMSCs were identified by BMSCs’ surface markers (CD29, CD90, CD44, and CD45), using flow cytometry. (C, D) The cytokine secretion and gene expression of IL-6 and TNF-α derived from BMSCs under different concentrations of LPS stimulation were measured by ELISA and RT-qPCR. (E) The cell viability of BMSCs under treatment with different concentrations of LPS was assayed by CCK-8 and (F) the cell death was determined with the help of Annexin V-FITC/PI dual staining by flow cytometry. *P<0.05 versus 0 ng/mL group.

**
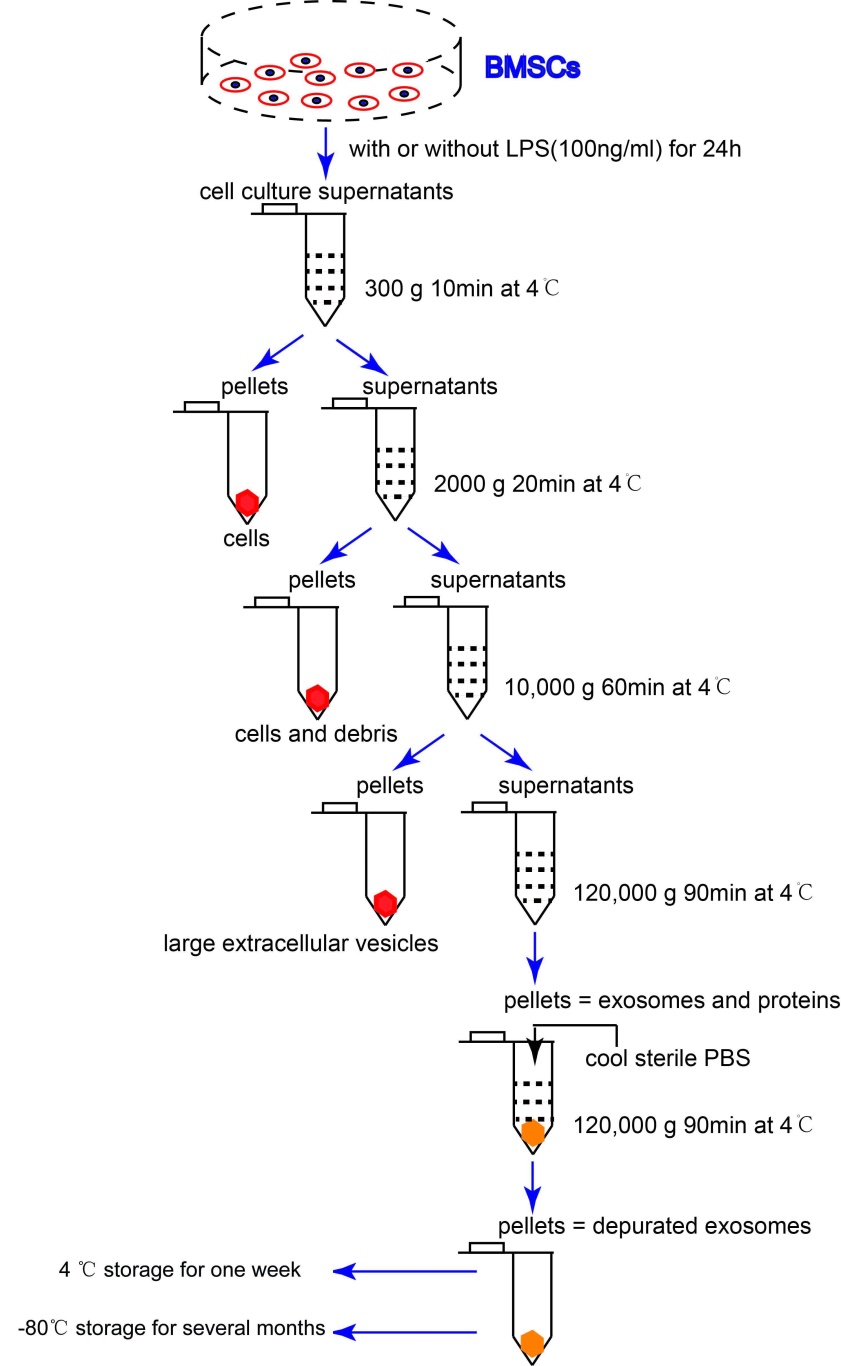
**

**Fig. S2. Isolation and purification of BMSCs-derived exosomes.** Briefly, after cell culturing, conditioned culture medium supernatants were differentially and sequentially centrifuged at 300 xg for 10 min and at 2000 xg for 20 min to discard any cells and debris, following at 10,000 xg for 60 min to remove large extracellular vesicles, and then centrifuged at 120,000 xg for 90 min at 4 ℃ to precipitate exosomes. To obtain depurated exosomes, the precipitates were washed once with cold, sterile PBS at 120,000 xg for 90 min at 4 ℃. Finaly, the exosomal precipitates were suspended in PBS or dissolved in lysis buffer used for the subsequent analysis.

**
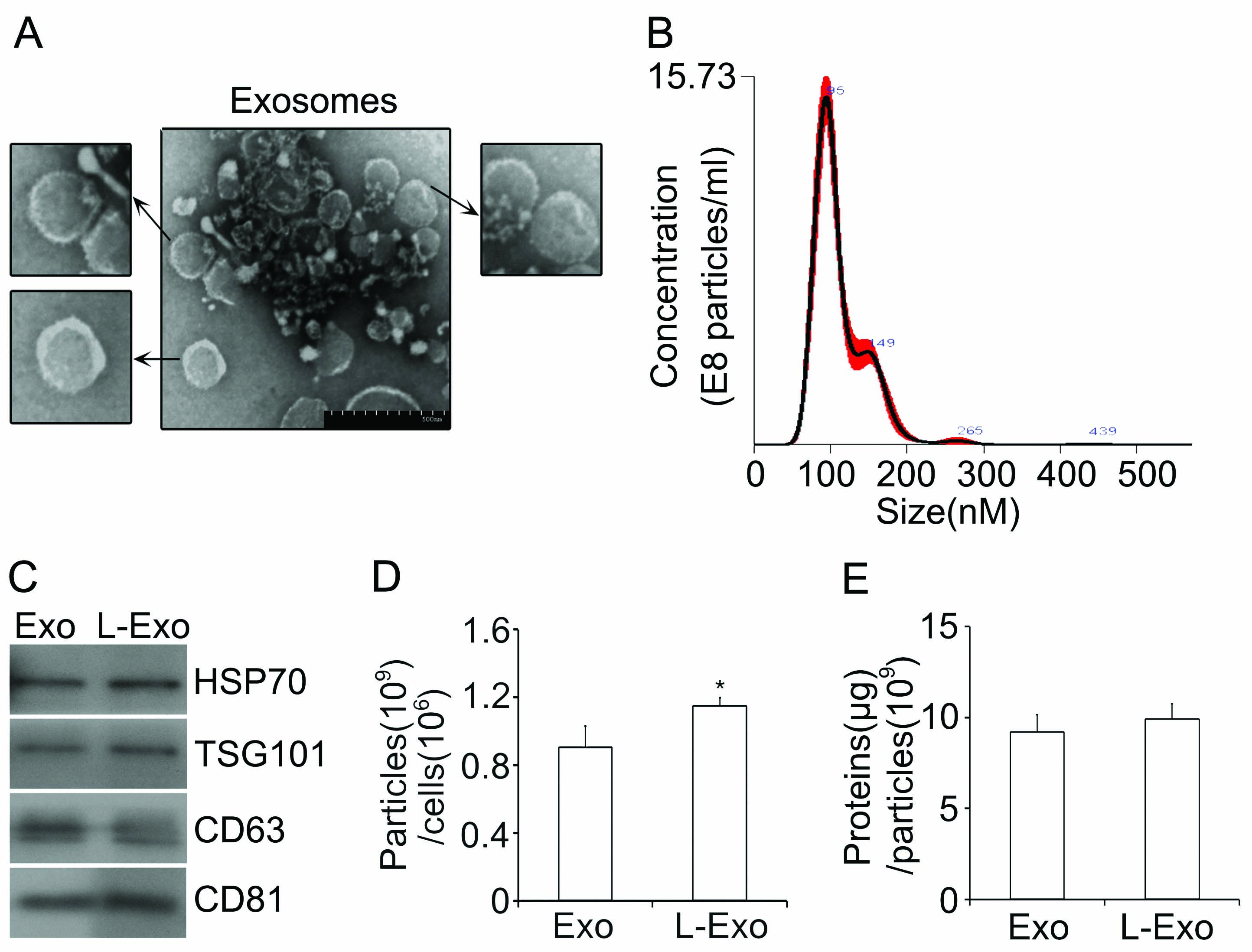
**

**Fig. S3.** **Characterization of BMSCs-derived exosomes.** (A) The typical morphology of exosomes was determined by transmission electron microscopy (TEM). The arrows pointed the representative exosomes. (B) The size of exosomes was measured by nanoparticle tracking analysis (NTA). The mean diameter of these particles is 111.8 ± 0.7 nm, and the mode diameter of these particles is 94.6 ± 2.2 nm. (C) The exosomal protein markers (CD63, CD81, TSG101, and Hsp70) were detected by Western blot analysis. (D) The particle concentration of exosomes was detected by NTA. (E) The protein concentration of exosomes was measured by BCA. Exo, exosomes derived from BMSCs; L-Exo, exosomes derived from BMSCs with LPS preconditioned. *P<0.05 versus Exo group.

**
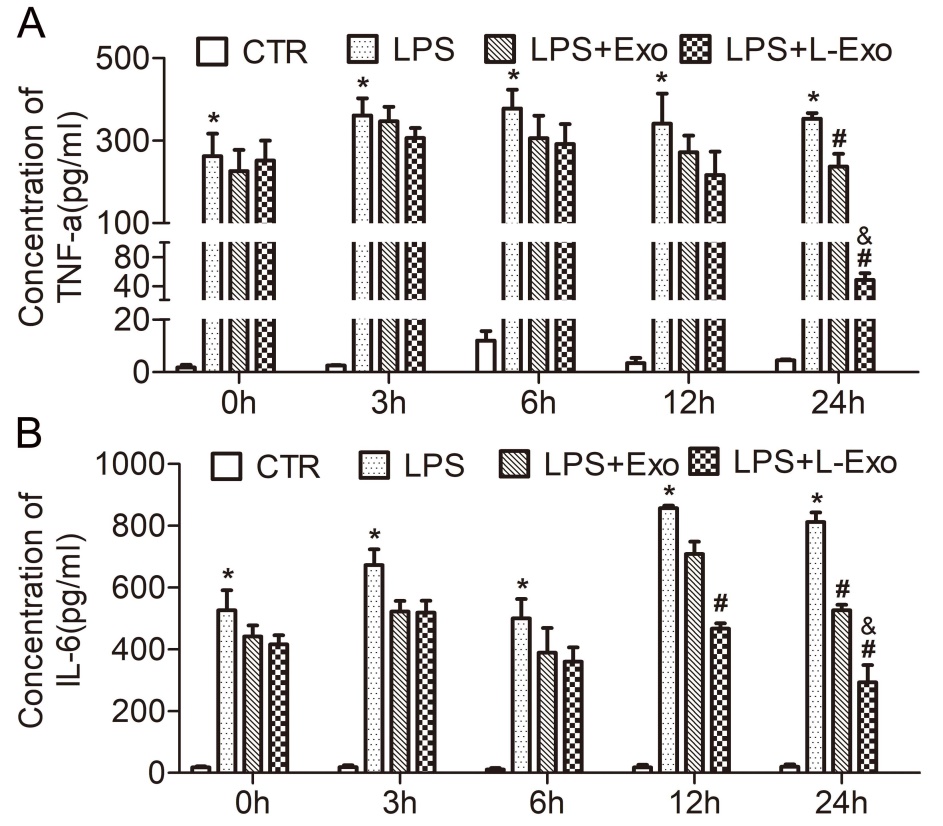
**

**Fig. S4. The co-culture of Raw264.7 cells with Exo and L-Exo in advance attenuated inflammation in a time-dependent manner.** Before treated with LPS (100 ng/ml) for 24 h, Raw264.7 cells were co-cultured with Exo and L-Exo at dose of 10 μg/ml for o h, 3 h, 6 h, 12 h, 24 h in advance. Then the cell medium supernatants were collected and the cytokine secretions of TNF-α (A) and IL-6 (B) were measured by ELISA. *P<0.05 versus CTR group, ^#^P<0.05 versus LPS group, ^&^P<0.05 versus Exo group.


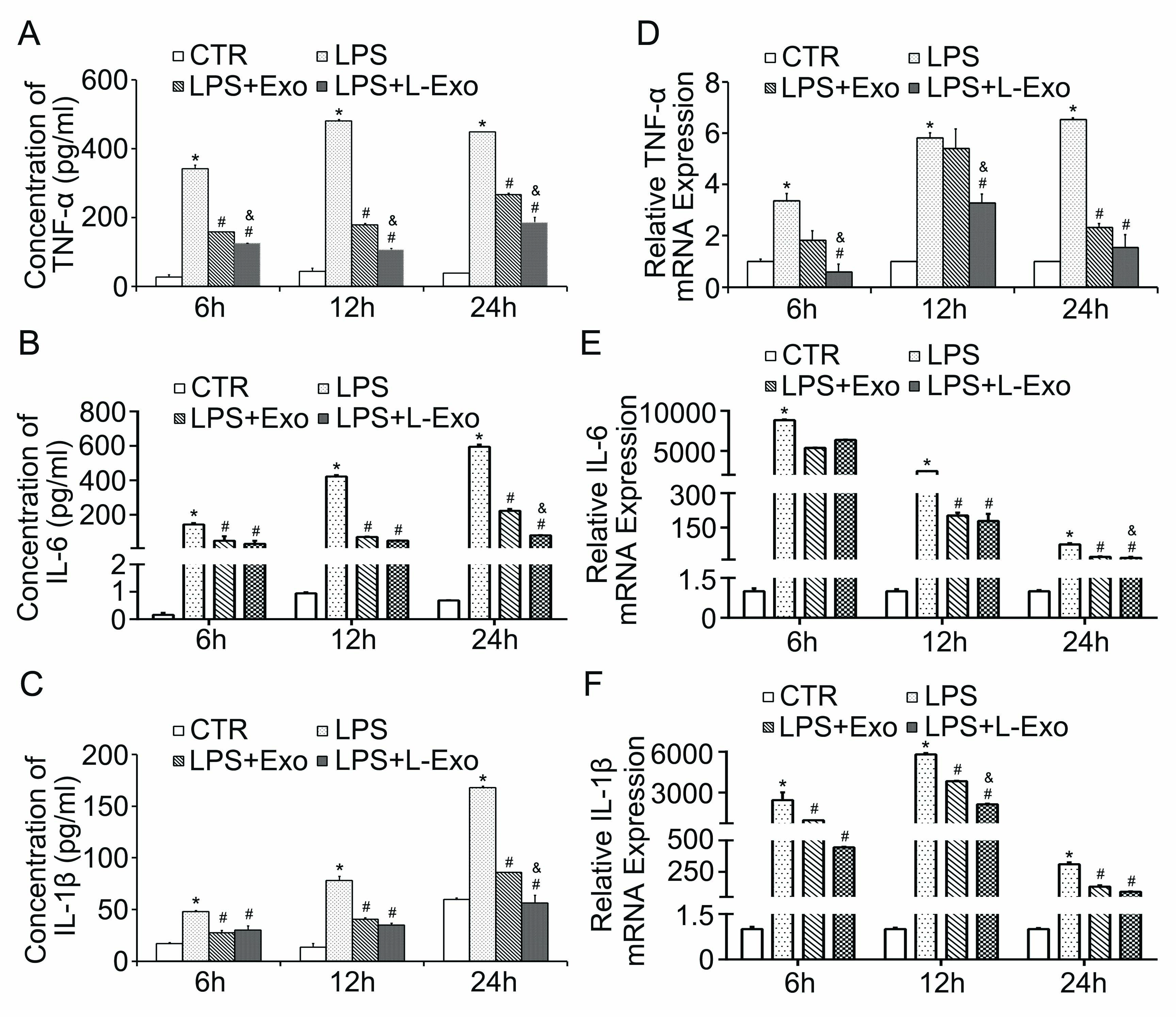


**Fig. S5. The co-culture of Raw264.7 cells with** **Exo and L-Exo** **at dose of 10 μg/ml** **under LPS** **stimulation** **at different time points (****6, 12, 24 h).** Following co-culture with Exo and L-Exo at dose of 10μg/ml for 24 h, Raw264.7 cells were treated with LPS (100 ng/ml) at different time points (6, 12, and 24 h). The cytokine secretions (A-C) and gene expression (D-F) of IL-6, TNF-α, and IL-1β were measured. *P<0.05 versus CTR group, ^#^P<0.05 versus LPS group, ^&^P<0.05 versus Exo group.

| Table S1 Primers used for RT-qPCR | | |
| --- | --- | --- |
| Gene | Forward primer | Reverse primer |
| Rat |  |  |
| TNF-α | TACTGAACTTCGGGGTGATCG | CCACTTGGTGGTTTGCTACG |
| IL-6 | GACTGATGTTGTTGACAGCCACTGC | TAGCCACTCCTTCTGTGACTCTAACT |
| IL-1β | GCTTCAGGCAGGCAGTATCA | TGCAGTTGTCTAATGGGAACG |
| IL-10 | CTTTCACTTGCCCTCATCC | ACAAACAATACGCCATTCCC |
| β-actin | ATTGTAACCAACTGGGACG | TCTCCAGGGAGGAAGAGG |
| Mouse |  |  |
| TNF-α | GCCACCACGCTCTTCTGTCTAC | GGGTCTGGGCCATAGAACTGAT |
| IL-6 | CACATGTTCTCTGGGAAATCG | TTGTATCTCTGGAAGTTTCAGATTGTT |
| IL-1β | ACCTTCCAGGATGAGGACATGA | CTAATGGGAACGTCACACACCA |
| IL-10 | GGTTGCCAAGCCTTATCGGA | ACCTGCTCCACTGCCTTGCT |
| Arg I | TGGCTTTAACCTTGGCTTGCTTCG | AAAGAACAAGCCCTTGGGAGGAGA |
| iNOS | CTGCTGGTGGTGACAAGCACATTT | ATGTCATGAGCAAAGGCGCAGAAC |
| AKT1 | CCACCTGTCTCTAGGGTCCA | CATGGGACACAGCAACAAAC |
| AKT2 | TGGACCACAGTCATCGAGAG | CTTGTAATCCATGGCGTCCT |
| β-actin | GTGCTATGTTGCTCTAGACTTCG | ATGCCACAGGATTCCATACC |
